# Supplementary material for: Energy-loss return gate via liquid dielectric polarization
Source: Nat Commun. 2018 Apr 12;9:1437. doi: 10.1038/s41467-018-03893-7 (PMC5897353; doi:10.1038/s41467-018-03893-7)
Supplement: Supplementary file 1 — Supplementary Information [file 41467_2018_3893_MOESM1_ESM.pdf]

Energy-Loss Return Gate via Liquid Dielectric Polarization

Taehun Kim+, Hyungseok Yong+, Banseok Kim, Dongseob Kim, Dukhyun Choi, Yong Tae Park, and Sangmin Lee** **(+Equal contribution)** (***Corresponding author)**

Affiliations

T. Kim, H. Yong, B. Kim, Prof. S. Lee
School of Mechanical Engineering
Chung-Ang University
84, Heukseuk-ro, Dongjack-gu, Seoul 156-756, KOREA
E-mail: slee98@cau.ac.kr

Dr. D. Kim
Aircraft System Technology Group
Korea Institute of Industrial Technology
57, Yangho-gil, Yeongcheon-si, Gyeongbuk-do, 38822, KOREA

Prof. D. Choi
Department of Mechanical Engineering
Kyung Hee University
1732, Deogyeong-daero, Giheung, Yongin, Gyeonggi 446-701, KOREA

Prof. Y. T. Park
Department of Mechanical Engineering
Myongji University
116, Myongji-ro, Cheoin-gu, Yongnin, Gyeonggi 17058, KOREA
E-mail: ytpark@mju.ac.kr

Supplementary Figure

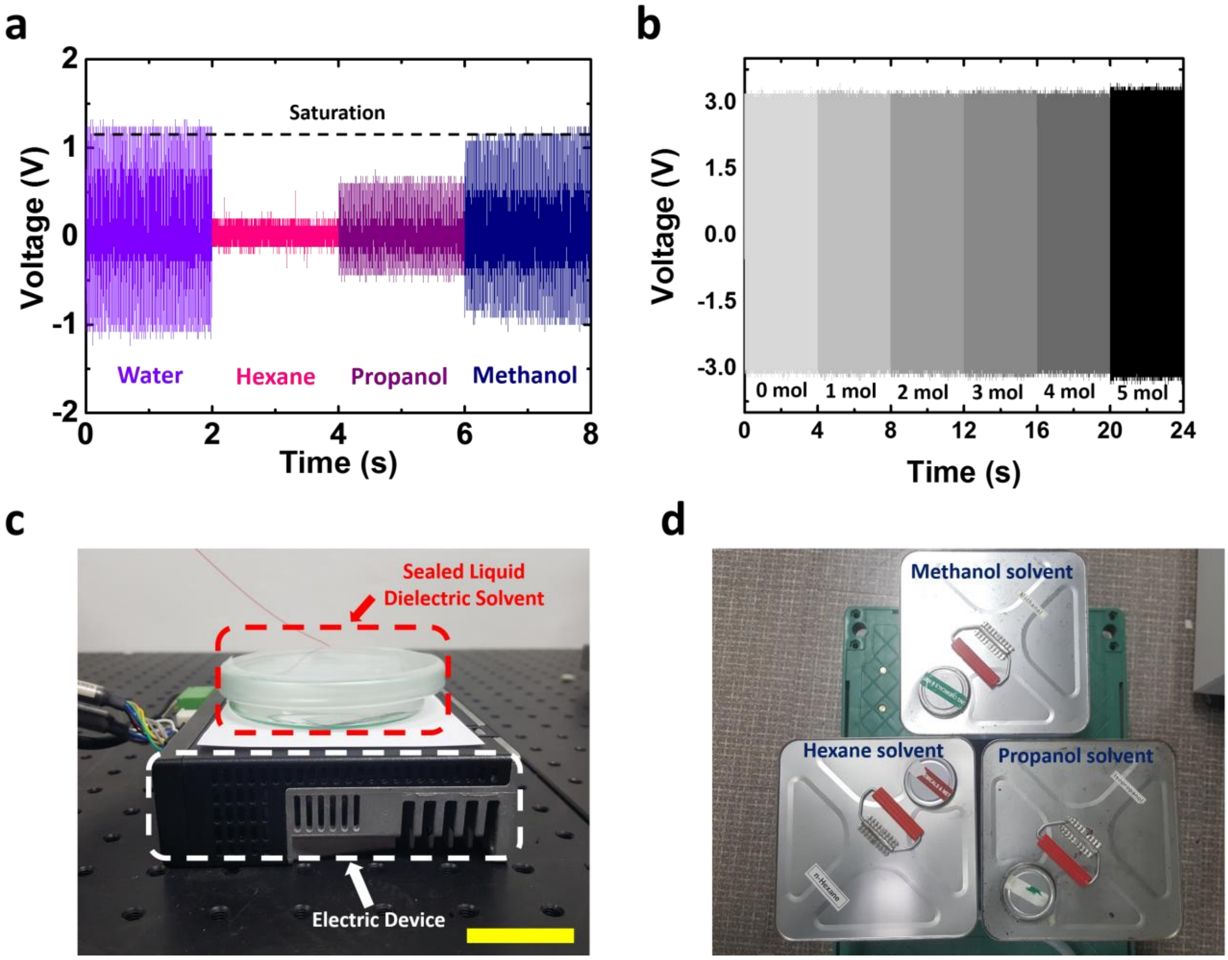

Supplementary Figure 1 ELRG electric outputs under various liquid dielectric properties. Electric device is plugged into a 60-Hz AC input electrical source. **a.** ELRG open-circuit voltage output with four different liquid materials. Dashed black line represents the saturation point. **b.** ELRG open-circuit voltage output under different ion concentrations. **c.** Image of real experimental setup with sealed liquid dielectric solvent and electric device, with yellow bar representing 3-cm scale. **d.** Image of solvents introduced in the experiments

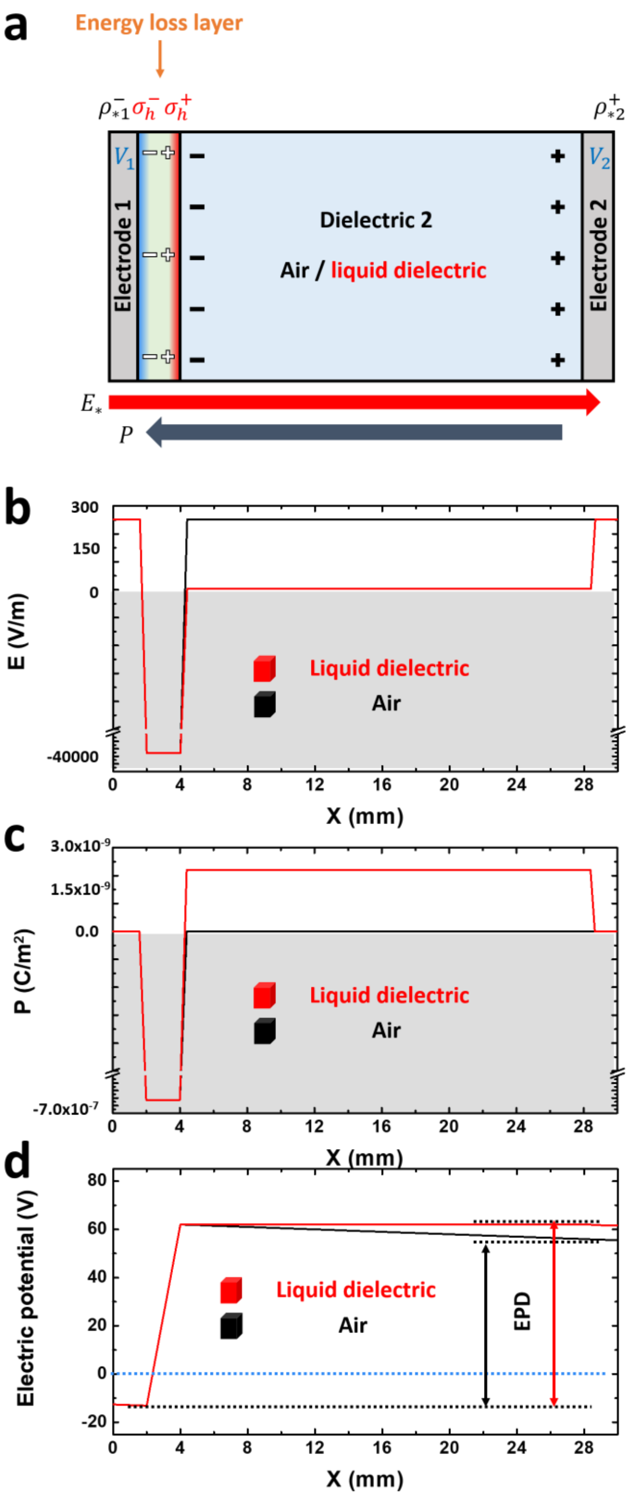

Supplementary Figure 2 Conformation and resultant of electrostatic simulation. a. Simulation scheme for calculating electric properties. **b.** Plot showing electric field distribution. **c.** Plot showing polarization distribution **d.** Plot showing electric potential distribution

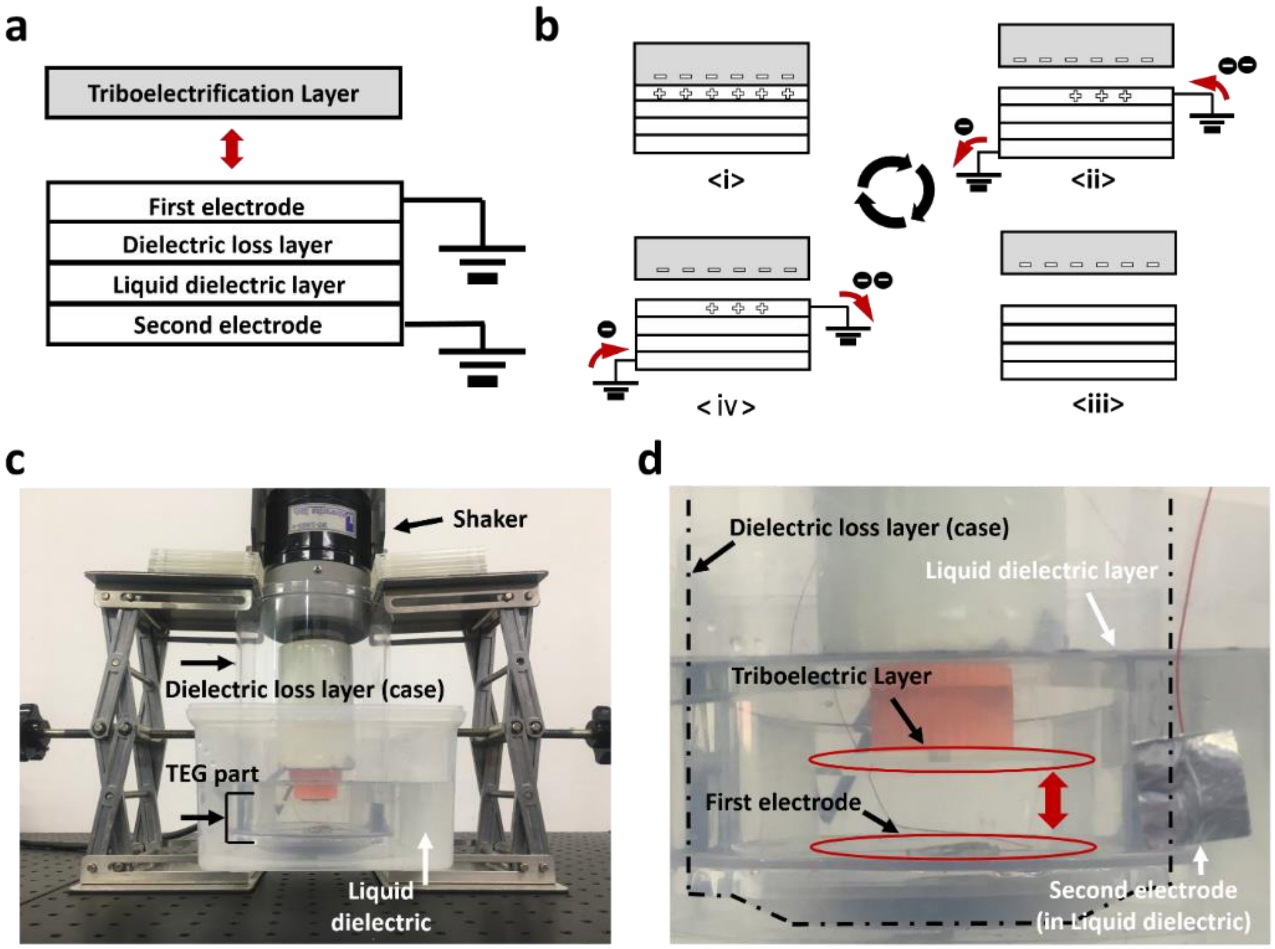

Supplementary Figure 3 Mechanism of triboelectric electricity generation. a. Conformation of a TEG. **b.** Schematic illustration showing step-by-step mechanism of a TEG. (i) Contact state; triboelectric layer and electrode are in charge equilibrium state; (ii) separation process (first electrode acquires free electrons and second electrode releases free electrons); (iii) separation state; triboelectric layer and electrode are in charge equilibrium state; and (iv) contact process (first electrode releases free electrons and second electrode acquires free electrons). **c.** Picture of real experimental setup of a TEG with a dielectric loss layer. **d.** Enlarged view of a TEG with ELRG setup

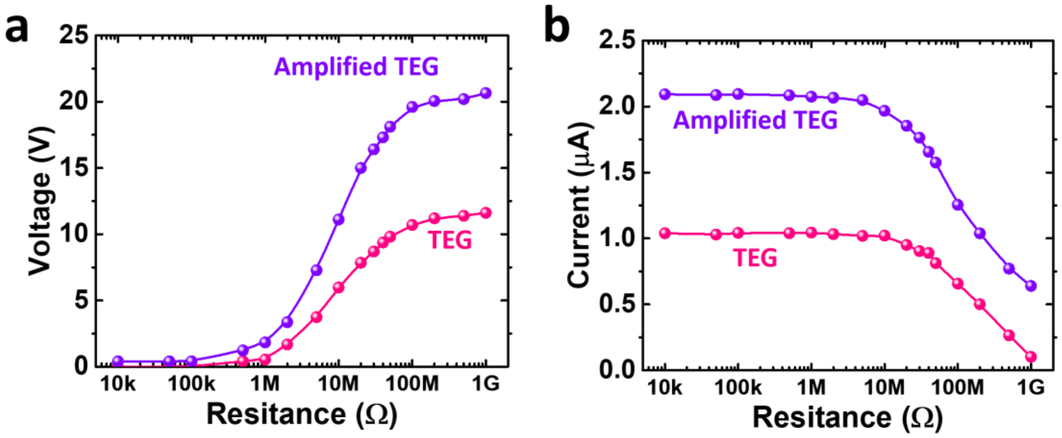

Supplementary Figure 4 Voltage and current outputs from TEG under load resistances. Load resistances are ranging from 10 kΩ to 1 GΩ. **a.** Plot showing voltage outputs. **b.** Plot showing current outputs

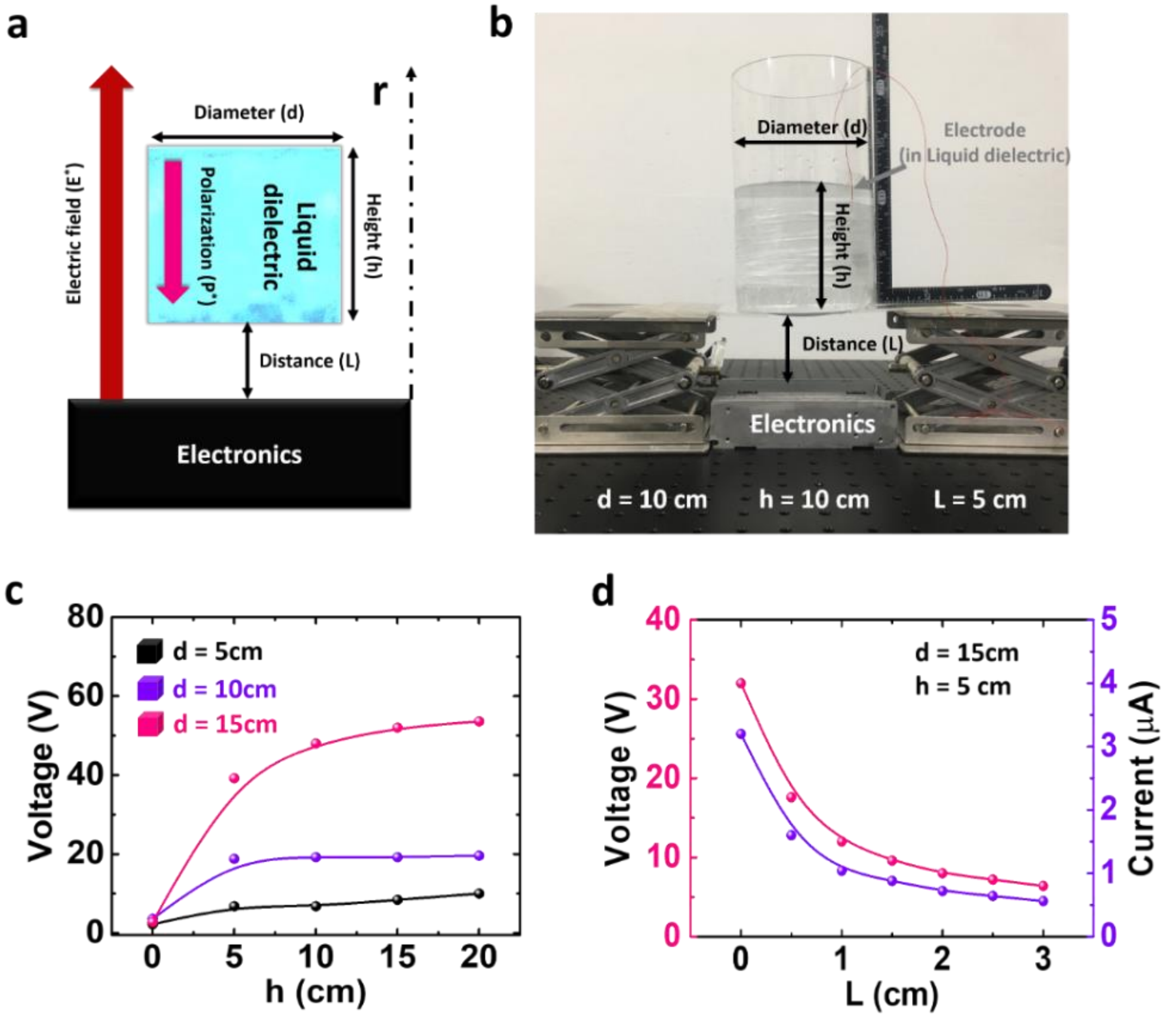

Supplementary Figure 5 Comparison of the ELRG electric outputs under the geometry of the liquid dielectric. a. Schematic illustration with geometric factors for measuring ELRG outputs. **b.** Image showing real setup under experimental condition of d = 10 cm, h = 10 cm, and L = 10. **c.** Plot showing voltage outputs under the consideration of sectional area and volume with L = 0. **d.** Plot showing voltage and current outputs under the consideration of distance between the liquid dielectric and electric system

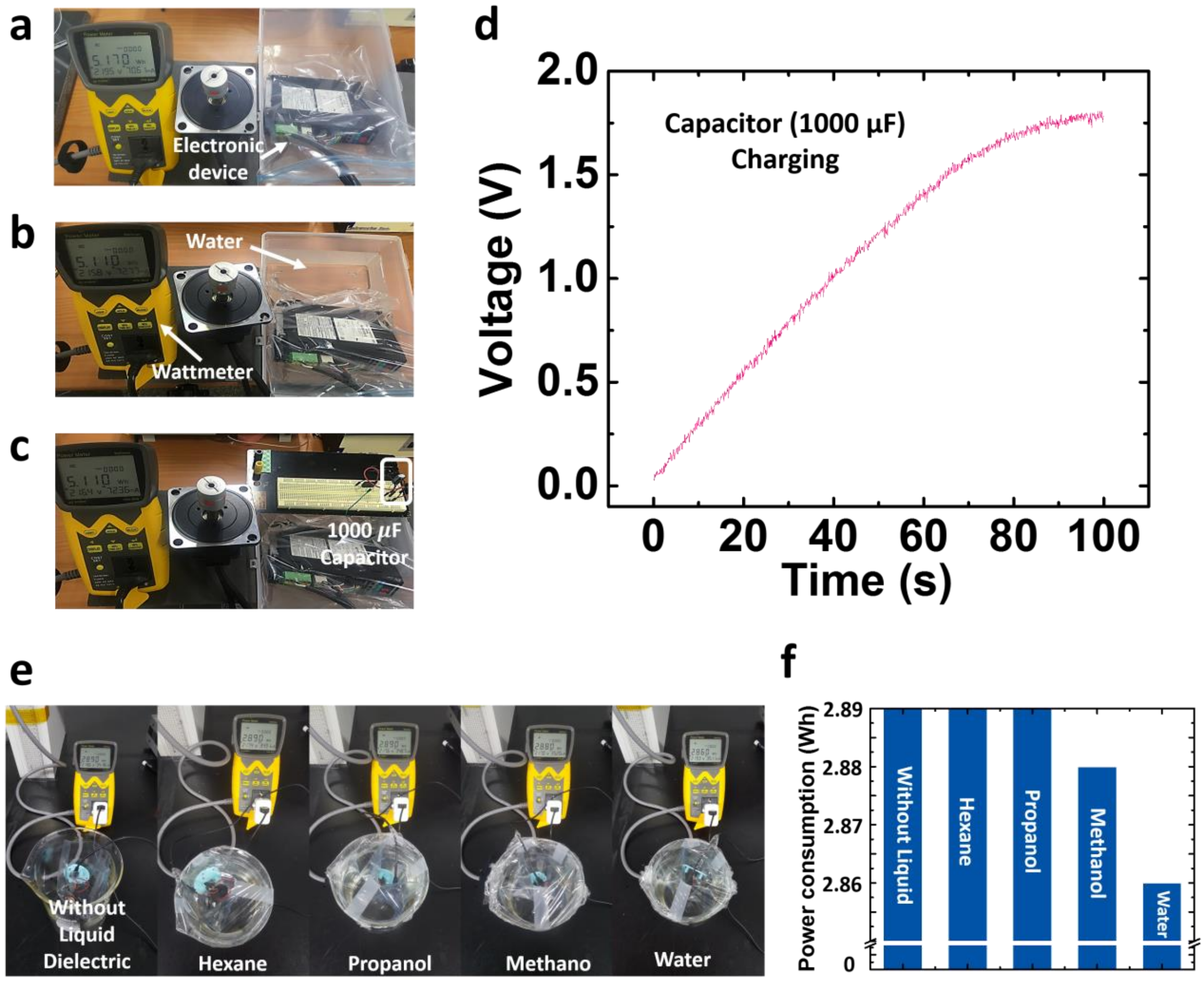

Supplementary Figure 6 Experiment configuration to demonstrate ELRG effect. **a.** Experiment pictures. Without liquid dielectric / without charging; **b.** with liquid dielectric / without charging; and **c.** with liquid dielectric / with charging. **d.** Plot showing capacitor charging. **e.** Electronic device power consumption depending on various liquid dielectric materials with different thermal conductivity. **f.** Plot showing reduced power consumption when various liquid dielectrics are integrated

.

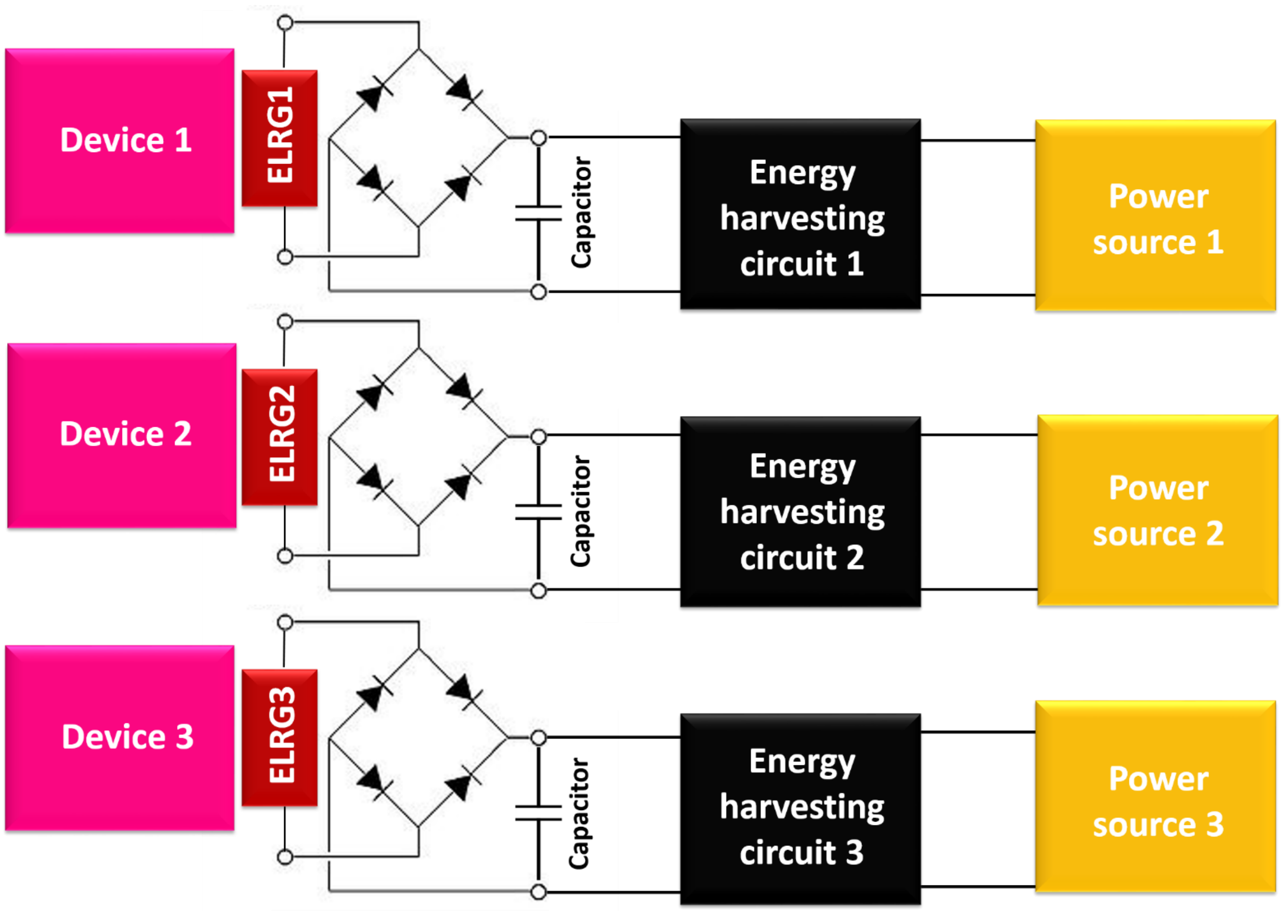

Supplementary Figure 7 Schematic illustration of constructive energy harvesting model. All electric devices are plugged into a 60-Hz AC input electrical source. Detailed electric outputs are given in Extended Data 8.

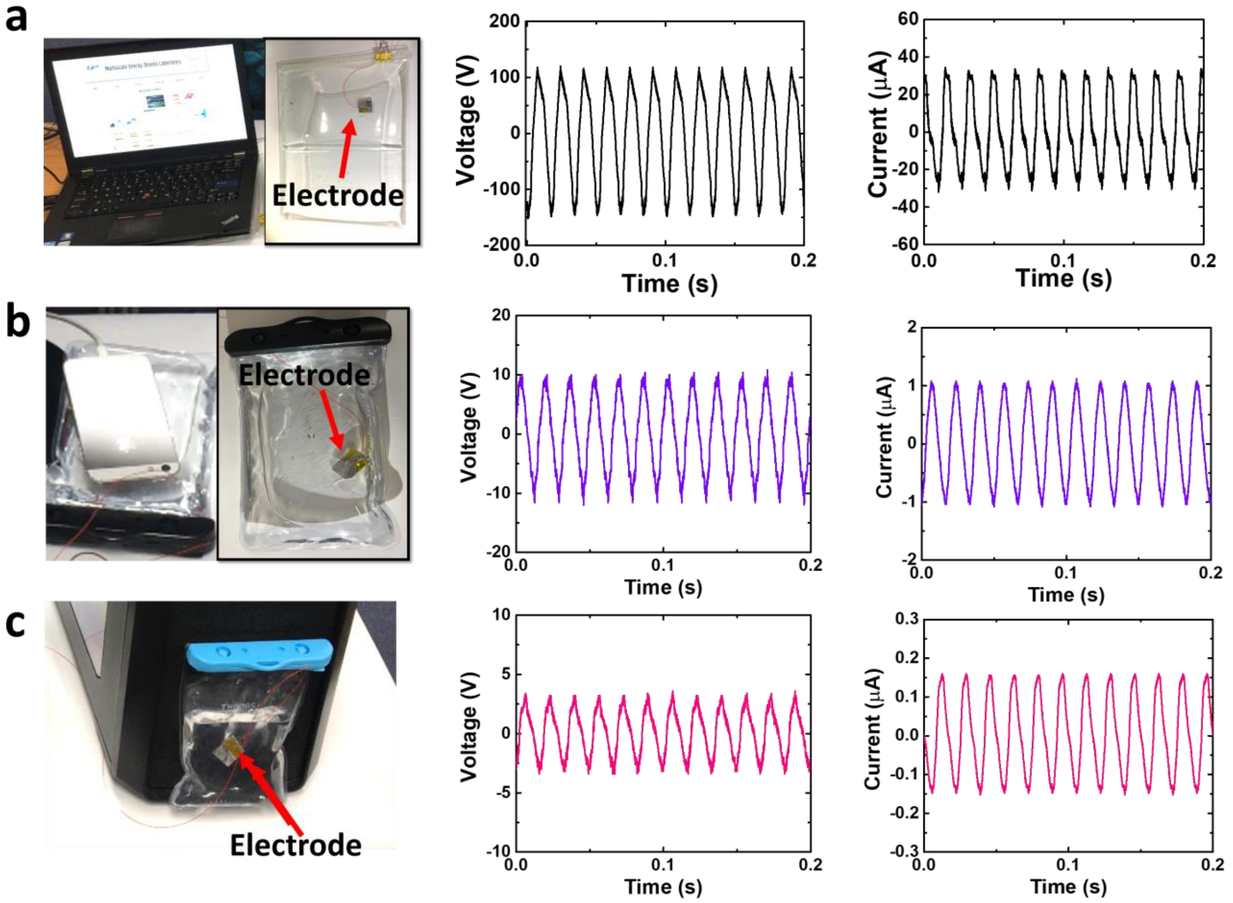

Supplementary Figure 8 Voltage and current outputs from respective ELRG. All electric devices are plugged into a 60-Hz AC input electrical source. **a.** Picture with ELRG-integrated laptop (left); plot showing open-circuit voltage (middle); short-circuit current (right). **b.** Picture with ELRG-integrated cell phone (left); plot showing open-circuit voltage (middle); short-circuit current (right). **c.** Picture with ELRG-integrated desk top (left); plot showing open-circuit voltage (middle); short-circuit current (right)

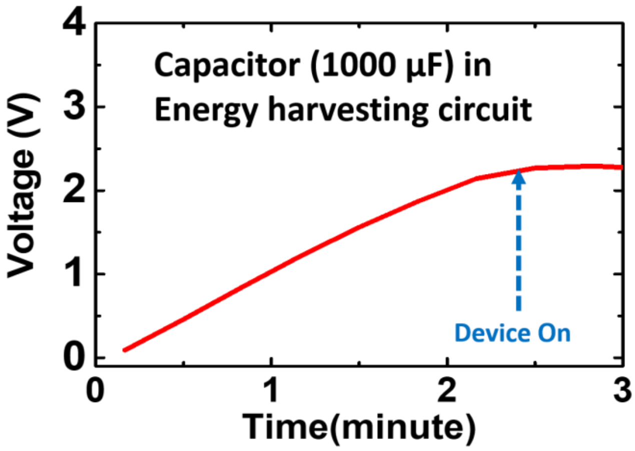

Supplementary Figure 9 Capacitor (1000 μF) charging plot from ELRG. The capacitor is connected to the other electric system when the plot line is saturated

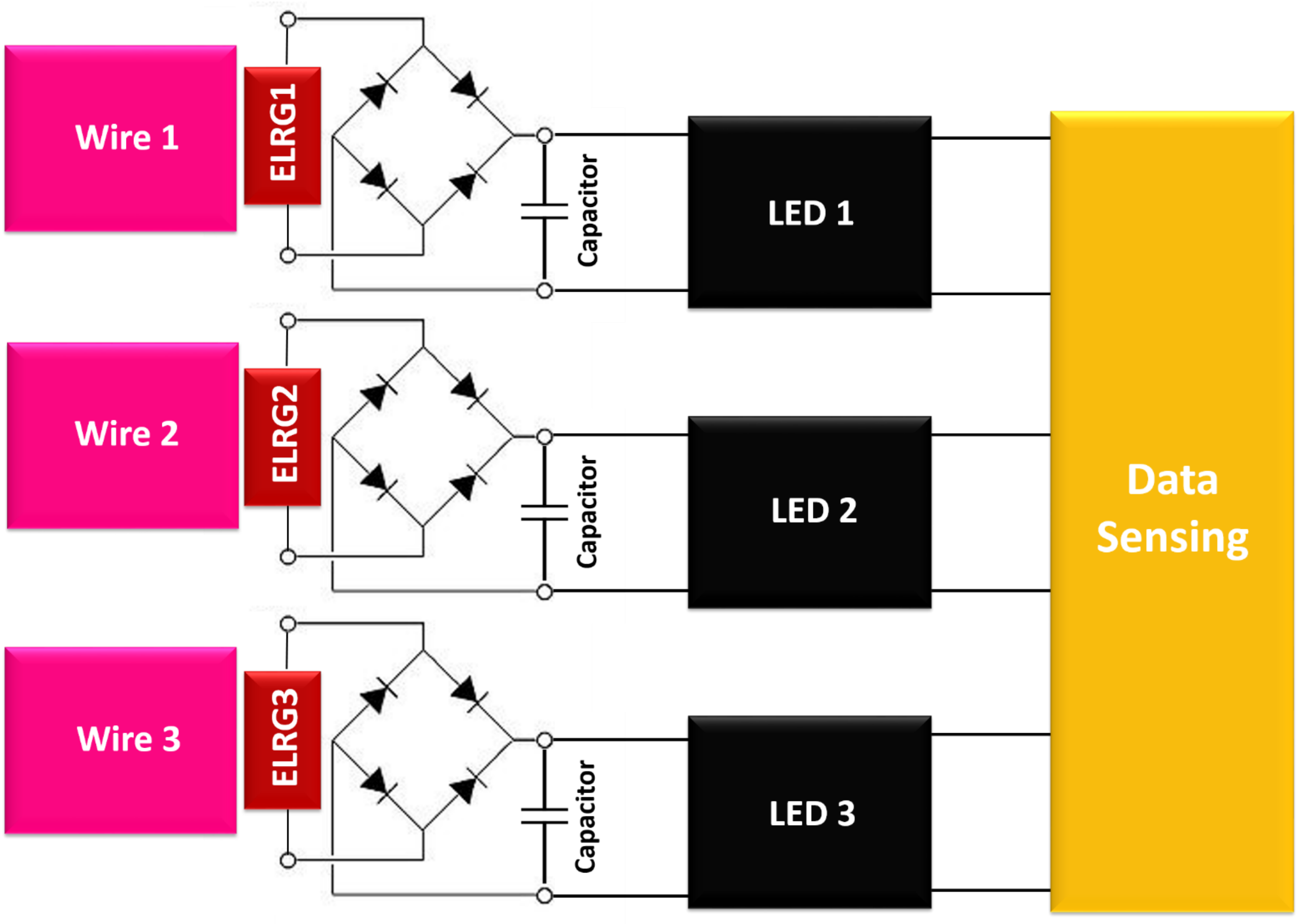

Supplementary Figure 10 Schematic illustration of constructive self-powered wire sensing model. All electric devices are plugged into a 60-Hz AC input electrical source
