# Supplementary material for: Energy-loss return gate via liquid dielectric polarization
Source: Nat Commun. 2018 Apr 12;9:1437. doi: 10.1038/s41467-018-03893-7 (PMC5897353; doi:10.1038/s41467-018-03893-7)
Supplement: Supplementary file 3 — Description of Additional Supplementary Files [file 41467_2018_3893_MOESM3_ESM.pdf]

Description of Additional Supplementary Files

File Name: Supplementary Movie 1
Description: ELRG application for our daily lives

File Name: Supplementary Movie 2
Description: ELRG application for self-powered sensing system
